# Supplementary material for: Dietary Supplementation throughout Life with Non-Digestible Oligosaccharides and/or n-3 Poly-Unsaturated Fatty Acids in Healthy Mice Modulates the Gut–Immune System–Brain Axis
Source: Nutrients. 2021 Dec 30;14(1):173. doi: 10.3390/nu14010173 (PMC8746884; doi:10.3390/nu14010173)
Supplement: Supplementary file 1 [file nutrients-14-00173-s001.zip › nutrients-1503129-supplementary.pdf]

Supplementary materials

Dietary supplementation throughout life with non-digestible oligosaccharides and/or n-3 poly-unsaturated fatty acids in healthy mice modulates gut-immune system-brain axis

K. Szklany, P. Engen, A. Naqib, Stefan J. Green, A. Keshavarzian, A. Lopez Rincon, C. J. Siebrand, M. A. P. Diks, M. van de Kaa, J. Garssen, L. M. J. Knippels, A. D. Kraneveld

Table S1. Diet composition of the experimental diets.

	Control<br>g/kg diet	scGOS:lcFOS<br>g/kg diet	n-3 PUFA<br>g/kg diet	Combination<br>g/kg diet
**Carbohydrates**				
Cornstarch	367.5	36.,5	367.5	367.5
Dextrinized cornstarch	122.5	122.5	122.5	122.5
Sucrose	91.0	91.0	91.0	91.0
Lactose monohydrate EF	9.5	0	9.5	0
Dextrose monohydrate	9.9	0	9.9	0
**Fiber**				
Cellulose	50.0	19.8	50.0	19.8
GOS sirop Vivinal (45% GOS)	0	60.0	0	60.0
Inulin HP (lcFOS) (97% FOS)	0	3.1	0	3.1
**Protein**				
Soy protein	200.0	200.0	200.0	200.0
DL-methionine	2.0	2.0	2.0	2.0
L-cystine	1.0	1.0	1.0	1.0
**Fat**				
Soybean oil	100.0	100.0	40.0	40.0
BioPure DHA IF tuna oil	0	0	60.0	60.0
**Others**				
Mineral mix AIN-93G	35.0	35.0	35.0	35.0
Vitamin mix AIN-93VX	10.0	10.0	10.0	10.0
Choline bitartrate	2.5	2.5	2.5	2.5
Tert-butylhydroquinone	0.014	0.014	0.014	0.014

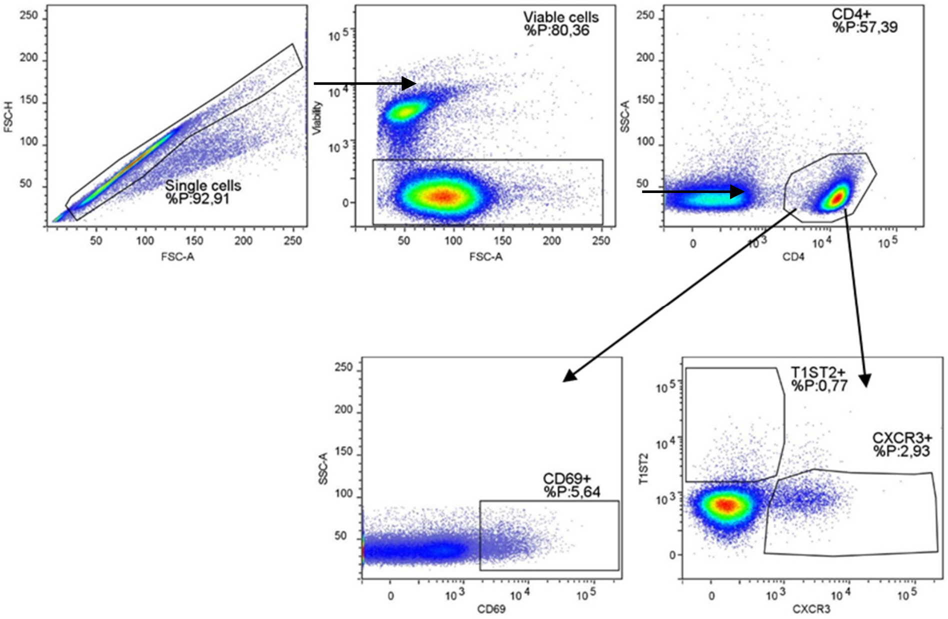

Figure S1. Gating strategy used in the flowcytometry analysis. First single cells were gated, then viable cells, followed by CD4⁺ cells. The activated (CD69⁺) cells as well as the Th1 (CXCR3⁺) and Th2 (T1ST2+) cells were gated from the CD4⁺ cells.

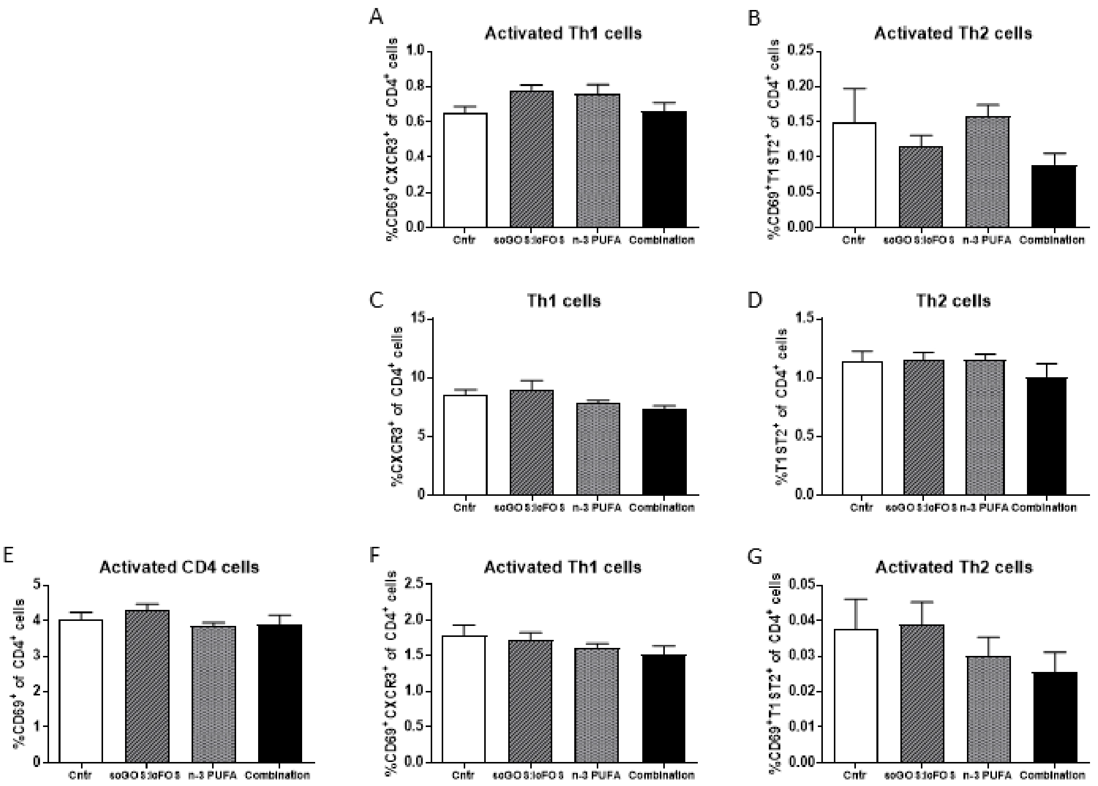

Figure S2. Th1 and Th2 response in MLN and spleen. (A,B) MLN: Activated Th1 and Th2 cells were not affected by the diets. (C-G) Spleen: Th1, Th2, activated T cells and activated Th1 and Th2 cells were unaffected by the diets. A-G: Data shown as mean +/- SEM. Analysed by 1-way ANOVA and Sidak multiple comparisons test. A-B: n = 6-10 mice per group, 2 samples in the control group, 3 samples in the n-3 PUFA group and 2 samples in the combination diet group excluded due to low number of viable cells. C-G: n=8-10 mice per group, 1 sample in the combination diet group excluded due to low number of viable cells. Th1: T helper 1. Th2: T helper 2.

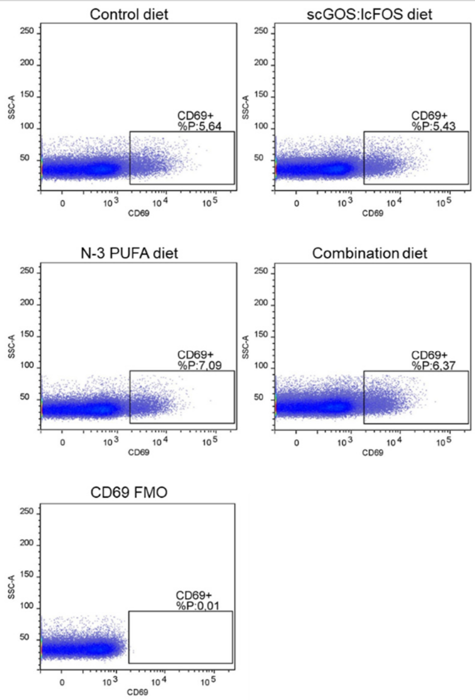

Figure S3. Representative dot plots of the CD69⁺ population from each diet group. The gate is based on the dot plot of the CD69 FMO.

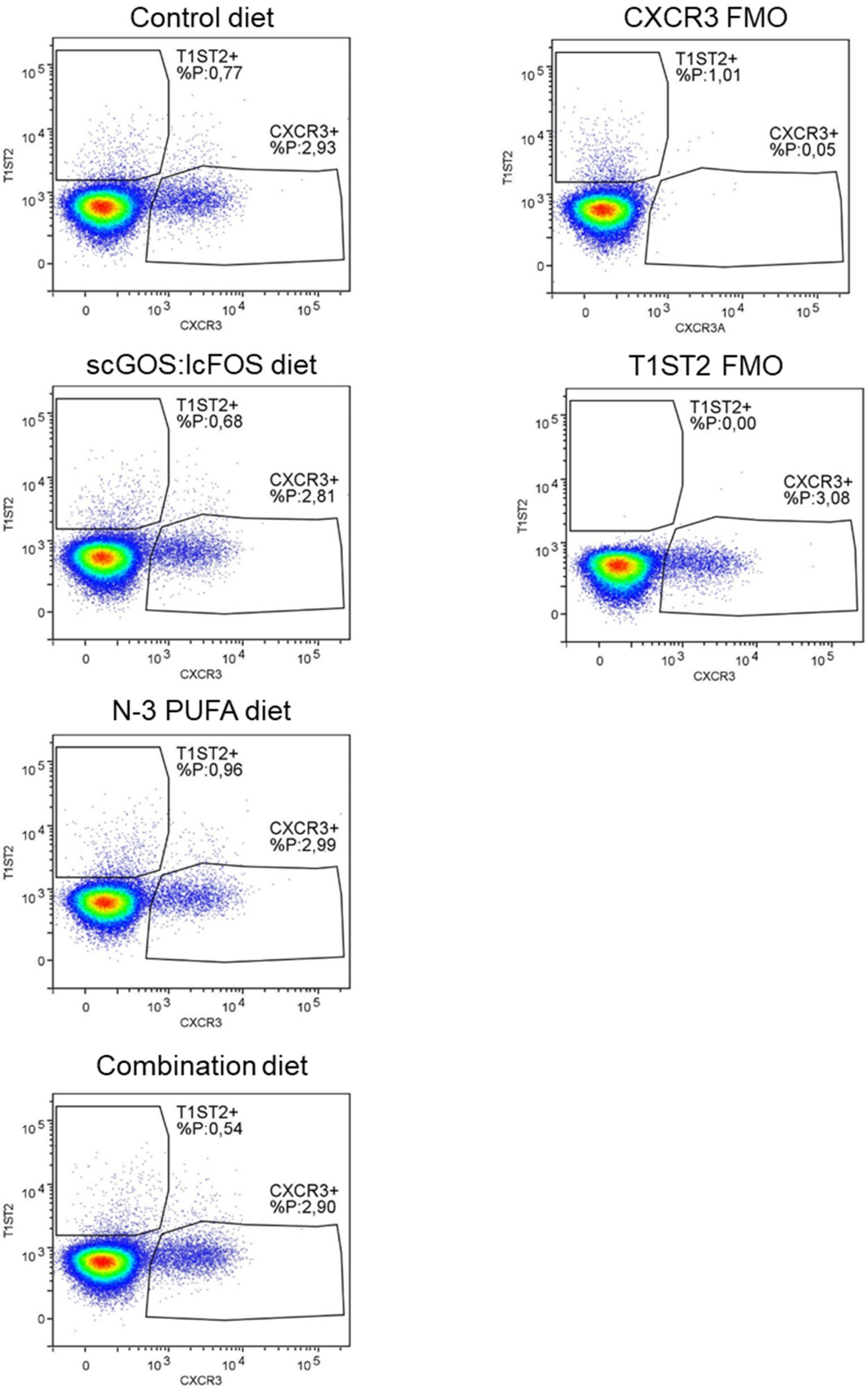

Figure S4. Representative dot plots of the Th1 (CXCR3+) and Th2 (T1ST2+) populations of each diet group. The gates were based on the CXCR3 FMO and T1ST2 FMO, respectively.

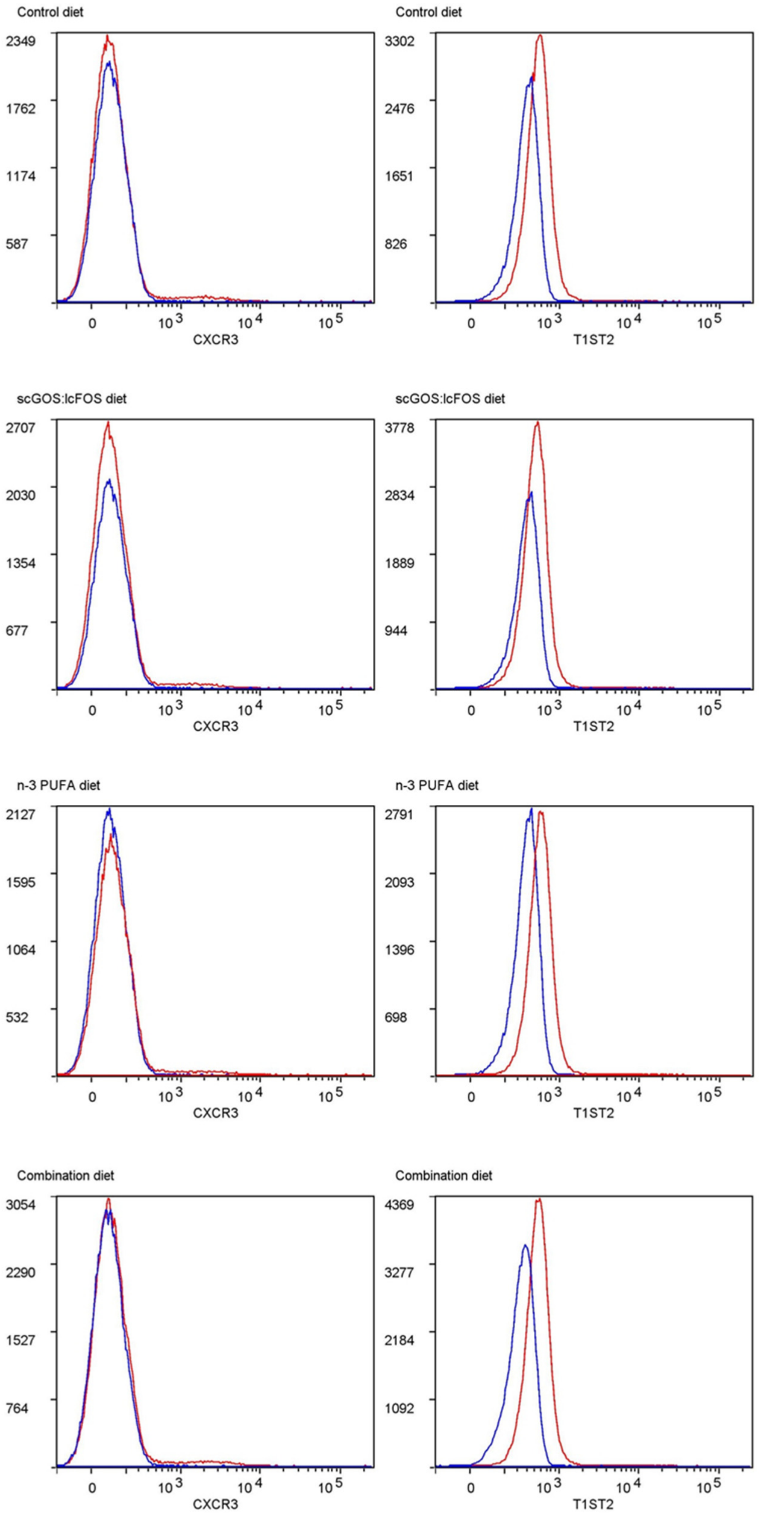

Figure S5. Representative histograms of CXCR3 and T1ST2 of each diet. The blue line indicates the FMO and the red line indicate the test sample.

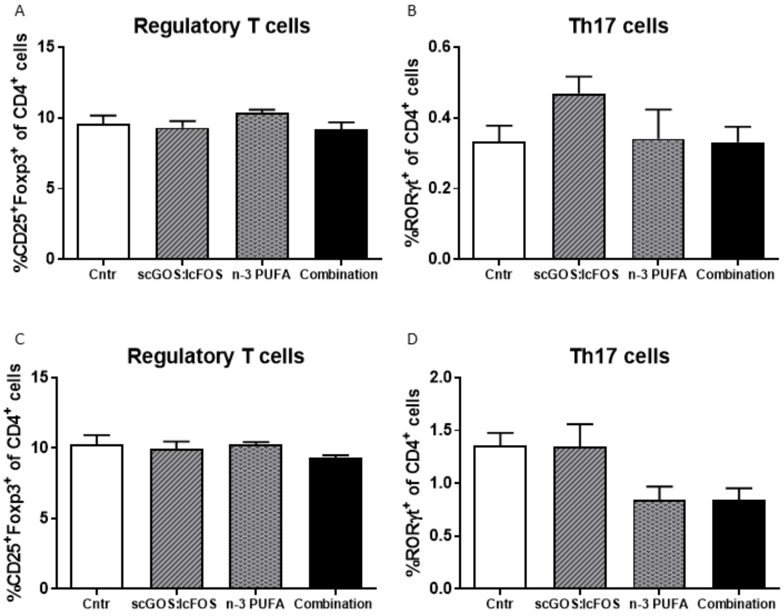

Figure S6. Treg and Th17 response in MLN and spleen. (A,B) No dietary effect on the Tregs and Th17 cells in the MLN. (C,D) No dietary effect on the Tregs and Th17 cells in the spleen. A-D: Data shown as mean +/- SEM. Analysed by 1-way ANOVA and Sidak multiple comparisons post-hoc test. n = 7-10 mice per group, in the MLN 1 sample in the control, n-3 PUFA and combination diet excluded due to low number of viable cells. Th1: T helper 1. Th2: T helper 2.

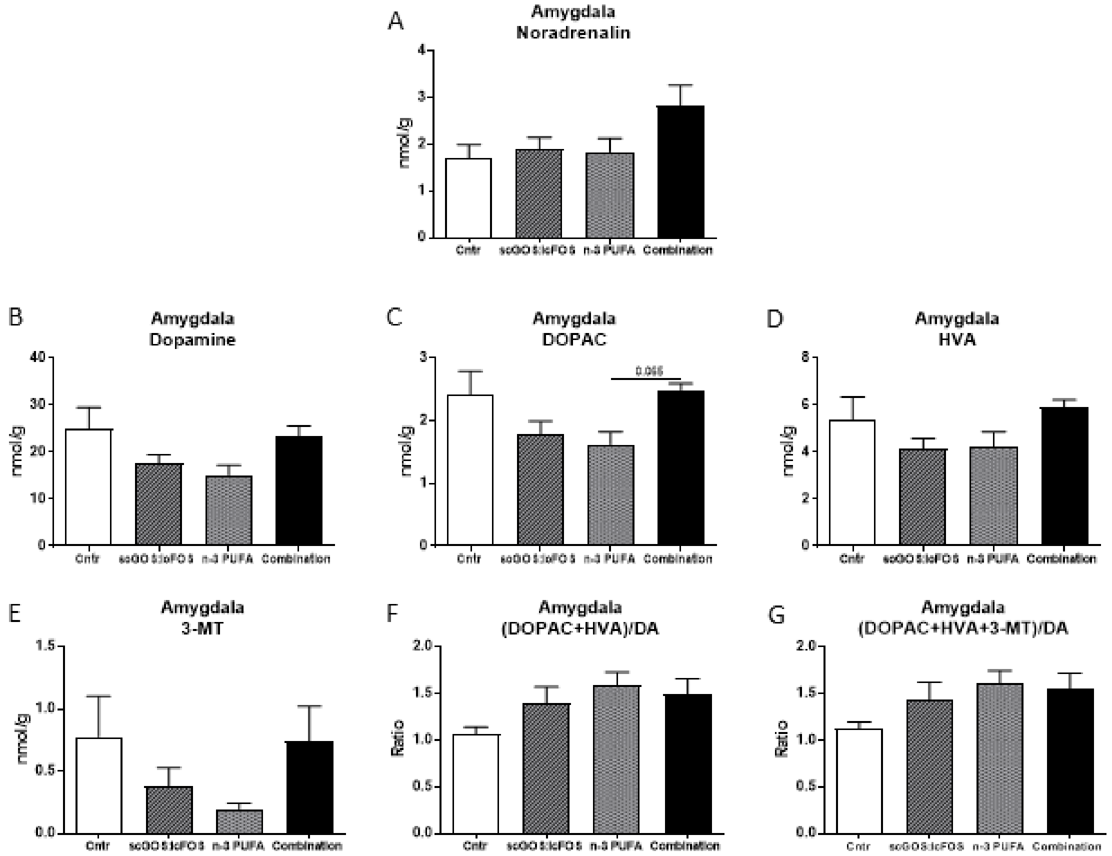

Figure S7. Monoamine levels in the amygdala. (A) Noradrenalin levels were unaffected by the diets. (B) Dopamine levels were unaffected by the diets. (C) The DOPAC level in the combination diet group tended to an increase compared to n-3 PUFA. (D,E) No dietary effect on HVA and 3-MT. (F,G) The turnover of dopamine was unaffected by the diets. A-G: Data shown as mean +/- SEM. Analysed by 1-way ANOVA and Sidak multiple comparisons post-hoc test. n = 4-5 samples per group, samples were pooled in pairs, in order to reach detection minimum, each sample contained two left brains. DOPAC: 3,4-dihydroxyphenylacetic acid. HVA: homovanillic acid. 3-MT: 3-methoxytyramine.

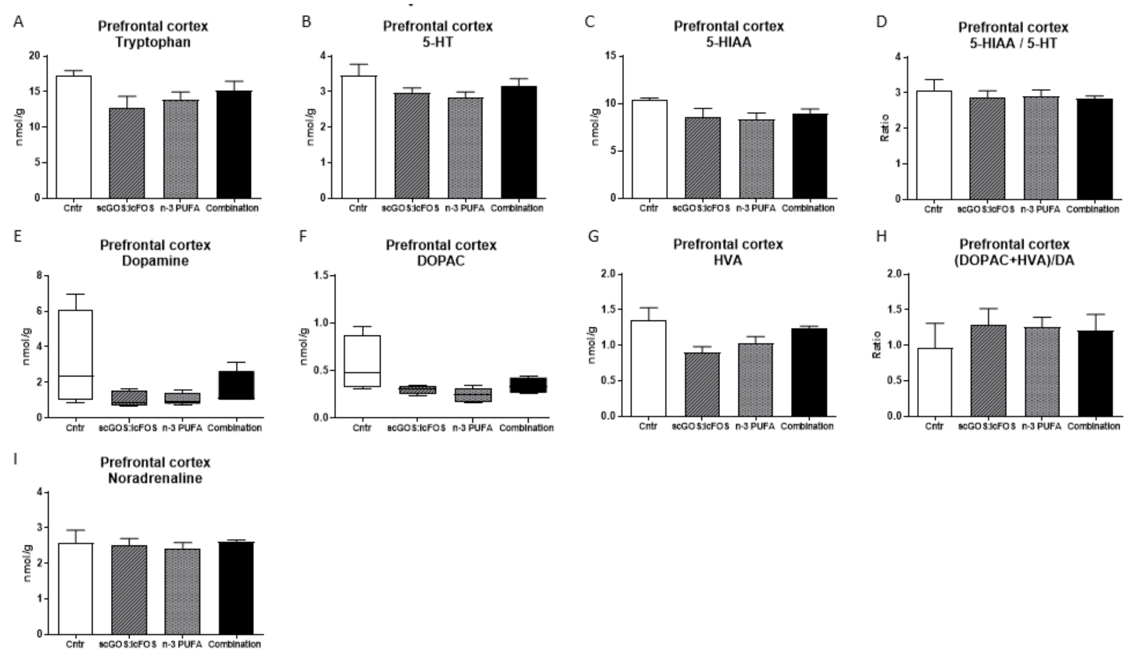

Figure S8. Monoamine levels and turnovers in the PFC. No differences were observed in any of the measured monoamines in the PFC. A-D,G-I: Data shown as mean +/- SEM. Analysed by 1-way ANOVA and Sidak multiple comparisons post-hoc test. E,F: Data shown as box-and-whiskers Tukey plot. Analysed by Kruskal-Wallis and Dunn's multiple comparisons post-hoc test. n = 4-5 samples per group, samples were pooled in pairs, in order to reach detection minimum, each sample contained two left brains. 5-HT: Serotonin. 5-HIAA: 5-hydroxyindoleacetic acid. DOPAC: 3,4-dihydroxyphenylacetic acid. HVA: homovanillic acid.

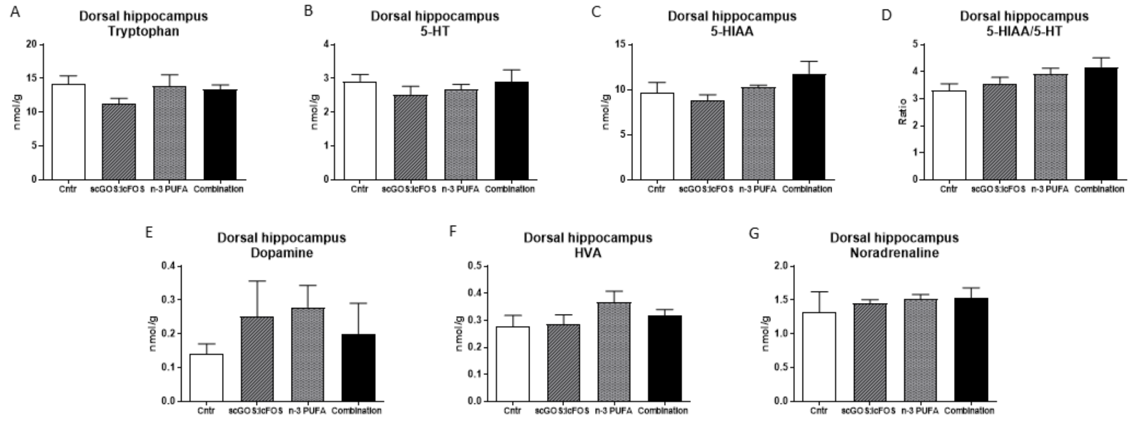

Figure S9. Monoamine levels and serotonin turnover in dorsal hippocampus. No differences were observed in any of the measured monoamines in the PFC. A-G: Data shown as mean +/- SEM. Analysed by 1-way ANOVA and Sidak multiple comparisons post-hoc test. n = 4-5 samples per group, samples were pooled in pairs, in order to reach detection minimum, each sample contained two left brains. 5-HT: Serotonin. 5-HIAA: 5-hydroxyindoleacetic acid. HVA: homovanillic acid.

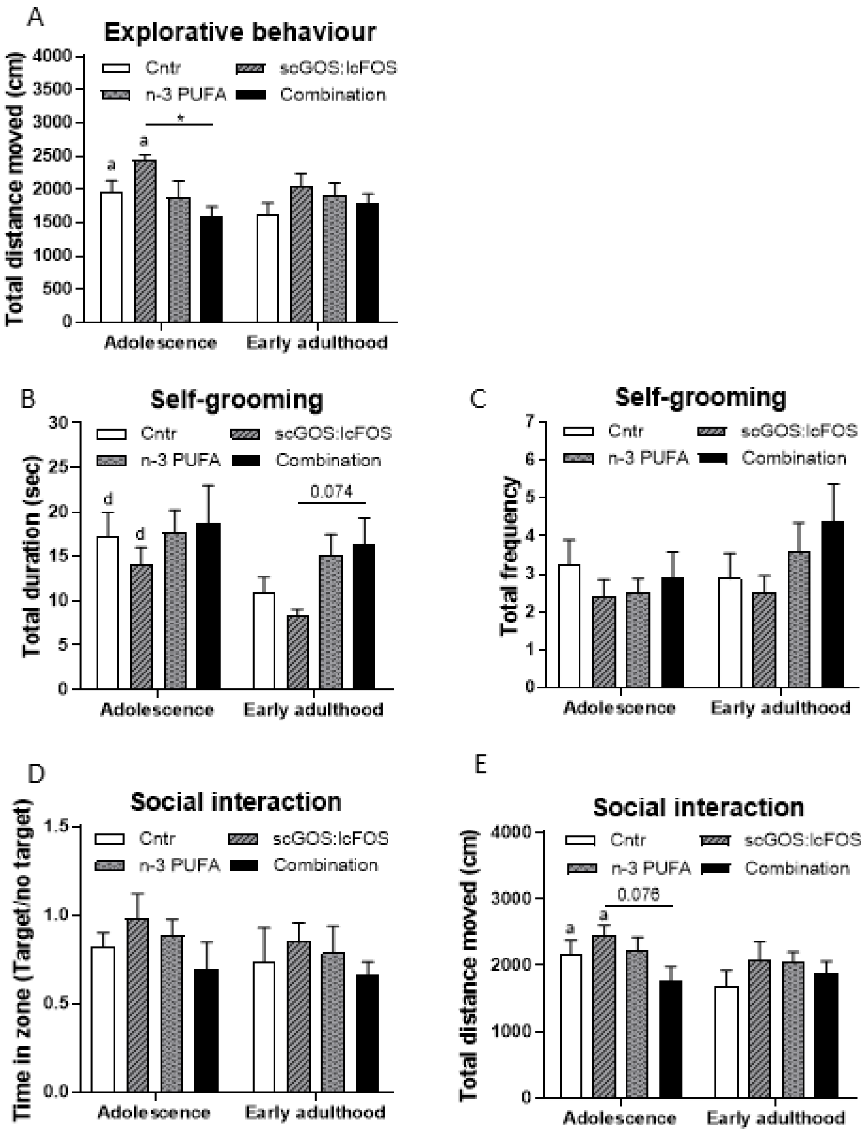

Figure S10. Behavioural data locomotion activity in the open field test, self-grooming and social interaction. (A) The mice receiving the control or the scGOS:lcFOS diet moved significantly less in early adulthood compared to adolescence. In adolescence, the locomotor activity was reduced in the combination diet group compared to the scGOS:lcFOS group. (B) The grooming duration tended towards a decrease overtime in the control as well as in the scGOS:lcFOS group. In early adulthood grooming duration showed an increasing trend in the combination group compared to the scGOS:lcFOS group. C: The grooming frequency showed no significant differences. D: The social interaction, shown as relative time in zone (target / no target), was neither affected by age nor diet. E: The locomotor activity (distance moved) in the control and scGOS:lcFOS receiving mice was significantly decreased over time. In adolescence, the locomotor activity tended towards a reduction in the combination diet group compared to the scGOS:lcFOS group. A-E: Data shown as mean +/- SEM. Analysed with mixed models, controlled for repeated measures, litter effect and Sidak's multiple comparisons post-hoc test. a = * $P < 0.05$ compared with early adulthood within diet group, b = ** $P < 0.01$ compared with early adulthood within diet group, c = *** $P < 0.001$ compared with early adulthood within diet group. n = 8-10 mice per group.
